# The best evidence for alcohol screening and brief intervention in primary care supports efficacy, at best, not effectiveness: *You say tomāto, I say tomăto? That’s not all it’s about*

**DOI:** 10.1186/1940-0640-9-14

**Published:** 2014-08-27

**Authors:** Richard Saitz

**Affiliations:** 1 Department of Community Health Sciences, Boston University School of Public Health, Boston, MA, USA; 2 Clinical Addiction Research and Education Unit, Boston Medical Center and Boston University School of Medicine, Boston, MA, USA

## Abstract

The review related to this manuscript is available at http://www.ascpjournal.org/content/9/1/13.

## 

Several meta-analyses find that patients with nondependent, unhealthy, alcohol use (identified by screening) who are randomized to receive brief counseling subsequently report drinking less than those randomized to control groups [1-4]. Most studies do not have biological confirmation, and those that do generally do not find effects on such outcomes, nor on any hard outcomes (though some do [4]). The lack of evidence for effects on these outcomes raises the possibility that alcohol screening and brief intervention efficacy remains unknown, since the modest effects on drinking could easily be due to social desirability bias [5] (i.e., patients are told they are in a study of counseling to reduce their drinking, they are randomly assigned to brief counseling with that goal, and then they are asked whether they reduced their drinking, so they may therefore report less drinking to please the interviewer; this would be expected to occur more often in an intervention than in a control group). This possibility should be resolved by further study (i.e., randomized trials with biological outcomes).

But if we take the positive trial findings as being valid—and many scientists and practitioners have [6]—the question is whether they represent efficacy or effectiveness. This question is important because if alcohol screening and brief intervention (ASBI) has efficacy, the next steps would be to determine how to implement it and retain effectiveness in real-world practice. But if the evidence supports effectiveness, little further study would be needed, and efforts would turn to dissemination and implementation.

In *Addiction Science & Clinical Practice*, Heather [7] makes the case that extant trials suggest that ASBI is effective. His case relies largely on two arguments. First and foremost, Heather observes that based on an effectiveness scale, randomized trials of ASBI can be characterized mainly as effectiveness studies. He does concede that the scale may have minor, and mainly methodological, limitations. But I believe the scale to be highly flawed for evaluating whether a trial of a preventive service like ASBI is an effectiveness or an efficacy study.

The scale as reported in Kaner et al. [4] emphasizes patients, practitioners, and intervention content. Such a scale could be useful for describing studies of lengthy interventions delivered by specialists developed for selected patients. Then, when such an intervention is somehow adapted for use in less selected patients to be delivered by generalists in general care settings, the trial will be appropriately characterized as an effectiveness study. But this conceptual approach does not apply to ASBI. ASBI was designed and developed as a brief service for all patients (i.e., unselected) to be delivered by practitioners who are not highly specialized, in general care settings. So even the most tightly controlled efficacy study would score as an effectiveness study on the scale.

More specifically, ASBI is designed for all primary care patients and starts with universal screening. It would not make sense to test a universal preventive service in selected or referred patients in an alcohol specialist clinic. If one did so, it would no longer be ASBI. By definition, any study of ASBI will score as effectiveness on “patients and problems” and “practice context”. Similarly any study of “brief” intervention will score high for effectiveness on “intervention content” because the timing could fit with general practice (e.g., 15 minutes). To be characterized as an efficacy study, the intervention would need to be much longer, but then of course it would no longer be brief intervention; it would be something else. ASBI based on personalized feedback and/or motivational interviewing is by design therapeutically flexible; so again, it will score high for effectiveness (by definition) since flexibility is characteristic of ASBI.

Thus, because of the nature of ASBI, just about any study of it would be characterized as an effectiveness study by the scale, and the only variability might be whether the ASBI or some component of it was performed by research staff, and whether it was monitored or supported by research staff. On these features, many ASBI studies in fact do look like efficacy studies. Practitioner training was usually implemented and orchestrated by researchers championing the effort. ASBI would not have occurred in the studies were it not for substantial researcher involvement. For example, researchers often performed the screening and provided the results to those conducting the BI. Investigators have been generally available to support ongoing ASBI implementation in these studies. And studies often monitored and assured fidelity of the BI. Although ASBI studies often begin with universal screening, many patients with unhealthy drinking are excluded for clinical and research reasons, such as drinking too little or too much, having alcohol dependence [8], having significant psychiatric or medical comorbidity, or for research reasons, such as being difficult to follow up, in addition to the fact that many simply choose not to participate in the trials.

The fact that ASBI trials have been conducted in primary care, often by primary care practitioners as interventionists in initially unselected patients, should not lead us to conclude that they are effectiveness trials, even if a scale would count them as such for those reasons. They are still studies that were controlled by researchers much more than usual interventions would be in routine clinical practice.

Evidence for the case that these have been largely efficacy studies is that two of the studies designed as effectiveness studies found no intervention effects [9-11]. They differed primarily from the rest of the ASBI trial literature in that the researchers had less control over intervention implementation (though even in the SIPS trial [9] when practitioners wouldn’t do the BI, researchers stepped in), fidelity, support, monitoring, and patient selection, yet they were similar to the rest of the literature in that they took place in primary care settings and used a flexible intervention delivered by real practitioners. These two studies clearly were effectiveness studies, very different from most other ASBI trials, yet they would score similarly on the effectiveness scale.

The second argument made by Heather [7] and Flay [12] is that in treatment effectiveness studies, the available intervention is optimized but acceptability could be variable, whereas the latter would be optimized in efficacy studies. But for ASBI, the distinction between these two (availability and acceptability) is minimal. Most ASBI intervention studies make BI “available in uniform fashion, within standardized contexts/setting, to a specified target group” (characteristic of efficacy studies according to Heather [7]). The distinguishing feature for Heather is whether or not it is delivered in a “manner acceptable to them [the patients]”. But once one has a patient who has agreed to be in a trial of an alcohol BI in primary care, it is likely that it will be acceptable (they will participate in the brief counseling), particularly if the intervention is flexible and provided on the spot, which ASBI usually is by design. ASBI as a service conceptualized and designed for primary care is brief, delivered in primary care at the time of the visit.

In sum, because of the very nature of the intervention, the only way to test ASBI is in studies that would have the characteristics of effectiveness studies according to the scale used by Kaner et al. [4]. I suggest that despite this, there are meaningful distinctions that should be made in characterizing these studies on the spectrum of efficacy versus effectiveness that are not captured by the scale for this preventive intervention. They are distinctions that primary care clinicians would instantly recognize—e.g., having special staff for training and implementation who are not usually in their practice taking steps to monitor and support fidelity, and having patients identified by research staff who prompt an intervention. Just because the study is completed in primary care and the intervention designed for that setting is (by definition) acceptable, does not make it an effectiveness study. Or, if we wish to say that based on the scale, they are effectiveness studies, then I suggest that for ASBI the distinction between effectiveness and efficacy studies is not meaningful. We then need another kind of study—a real-world [12] “does it work on a wet Wednesday in Wigan” study, which most ASBI studies are not.

If the current literature were truly replete with real-world ASBI effectiveness studies, then we would likely see ASBI being widely disseminated (it is not), and implemented successfully. Yet in one of the only large health systems with widespread ASBI implementation, the benefit of ASBI is undetectable [13]. Regardless of whether we agree to call ASBI studies effectiveness research (you say tomāto, I say tomăto), the bottom line is that the current literature is not very informative about whether ASBI works under real-world conditions.
